# Control of Locomotory Behavior of *Caenorhabditis elegans* by the Immunoglobulin Superfamily Protein RIG-3

**DOI:** 10.1534/genetics.119.302872

**Published:** 2019-11-18

**Authors:** Ashwani Bhardwaj, Pratima Pandey, Kavita Babu

**Affiliations:** * Department of Biological Sciences, Indian Institute of Science Education and Research (IISER) Mohali, Manauli 140306, India; † Centre for Neuroscience, Indian Institute of Science, Bangalore 560012, India

**Keywords:** RIG-3, AVA, GLR-1, neuropeptide, *Caenorhabditis elegans*

## Abstract

Cell surface immunoglobulin superfamily (IgSF) proteins play important roles in the development and function of the nervous system . Here we define the role of a *Caenorhabditis elegans* IgSF protein, RIG-3, in the function of the AVA command interneuron. This study reveals that RIG-3 regulates the abundance of the glutamate receptor subunit, GLR-1, in the AVA command interneuron and also regulates reversal behavior in *C. elegans*. The mutant strain lacking *rig-3* (*rig-3* (*ok2156*)) shows increased reversal frequency during local search behaviors. Genetic and behavioral experiments suggest that RIG-3 functions through GLR-1 to regulate reversal behavior. We also show that the increased reversal frequency seen in *rig-3* mutants is dependent on the increase in GLR-1 abundance at synaptic inputs to AVA, suggesting that RIG-3 alters the synaptic strength of incoming synapses through GLR-1. Consistent with the imaging experiments, altered synaptic strength was also reflected in increased calcium transients in *rig-3* mutants when compared to wild-type control animals. Our results further suggest that animals lacking *rig-3* show increased AVA activity, allowing the release of FLP-18 neuropeptide from AVA, which is an activity-dependent signaling molecule. Finally, we show that FLP-18 functions through the neuropeptide receptor, NPR-5, to modulate reversal behavior in *C. elegans*.

IN *Caenorhabditis elegans*, reversals play a vital role in defining locomotion-based behaviors (Zhao *et al.* 2003). Local search behavior is a locomotion-based behavior that is executed by *C. elegans* in the absence of food where they search for food locally (Zhao *et al.* 2003; Gray *et al.* 2005). Reverse movements have been studied in detail because reversals are critical factors that define the extent of local search (Zhao *et al.* 2003; Gray *et al.* 2005). Although the neural circuit that controls reversals is well defined, how this circuit is tuned during variable environmental conditions remains largely unknown. In the circuitry that controls reversals, the command interneurons, AVA, AVD, and AVE, are the control centers of reverse movement (Chalfie *et al.* 1985; Gray *et al.* 2005; Piggott *et al.* 2011). Hence, signaling through these sets of interneurons needs to be tightly regulated.

Glutamatergic transmission is predominant in the reversal circuitry where most of the sensory neurons that make either direct or indirect connection with reversal controlling command interneurons have been reported to release glutamate as a neurotransmitter (Choi *et al.* 2015). The command interneurons AVA, AVD, and AVE that are postsynaptic to these glutamatergic sensory neurons are known to express glutamate receptor subunits that include four non-NMDA (N-methyl-D-aspartate)-type glutamate receptors (GLR-1, GLR-2, GLR-4, and GLR-5) and two NMDA-type glutamate receptors (NMR-1 and NMR-2) (White *et al.* 1986; Brockie *et al.* 2001; Aronoff *et al.* 2004). Previous studies have reported several molecules in the reversal circuit that affect the abundance of glutamate receptors and hence control signaling via modulating synaptic strength to generate diverse reversal dependent behaviors. For example, the PDZ-domain containing protein LIN-10 regulates the synaptic localization of the GLR-1 receptors (Rongo *et al.* 1998). UNC-43, a calcium and calmodulin-dependent protein kinase II, is required to maintain the synaptic GLR-1 density, and SOL-1, a CUB domain protein, affects GLR-1 receptor function (Rongo and Kaplan 1999; Zheng *et al.* 2004, 2006).

In the nervous system, cell adhesion molecules are known to play essential roles in regulating synaptic structure and coordinating synaptic strength at neuronal junctions (Biederer *et al.* 2002; Rougon and Hobert 2003; Togashi *et al.* 2009). The immunoglobulin superfamily (IgSF) class of proteins is one of the most diverse classes of cell adhesion molecules that are known to play important roles in nervous system development and function, including synapse formation and function (Rougon and Hobert 2003). One such IgSF member, RIG-3, is expressed in the reversal circuitry neuron AVA, pharyngeal neurons I1, I4, M4 and NSM, and cholinergic motor neurons (Schwarz *et al.* 2009; Babu *et al.* 2011). Previous work has described the role of RIG-3 at the neuromuscular junction, where it has been shown to maintain the acetylcholine receptor ACR-16 (Babu *et al.* 2011; Pandey *et al.* 2017). However, although RIG-3 shows expression in the AVA command interneuron, its function in AVA remains largely unknown.

Here we show that RIG-3 functions to maintain the abundance of GLR-1 receptors in the AVA command interneuron and hence regulates the synaptic strength of AVA. This regulation of AVA function modulates AVA-dependent reversal behavior in *C. elegans*.

## Materials and Methods

### *C. elegans* strain maintenance

Strains were maintained on nematode growth medium (NGM) agar plates seeded with OP50 *Escherichia coli* at 20° under standard conditions (Brenner 1974). The *C. elegans* N2 Bristol strain was used as the wild-type (WT) control. All experiments were carried out with young adult hermaphrodites at ∼ 23°. A complete list of strains utilized in this study is given in Supplemental Material, Table S1. The primers used for genotyping different mutant strains are tabulated in Table S2. The N2 *C. elegans* and the OP50 *E. coli* ware obtained from the *Caenorhabditis* Genetics Center (CGC) (University of Minnesota, MN).

### Transgenic strains and constructs

Table S3 lists all the plasmids and constructs used in this study, Table S2 lists the primers used for constructing various plasmids. Plasmids were generated using a standard restriction digestion-based cloning strategy and sequenced before use in experiments. Briefly, promoters used in this study were cloned into pPD49.26 or pPD95.75, which includes P*flp-18* (4.1 kb), P*rig-3* (3 kb), P*gcy-5*, and P*gpa-3*. The complementary (cDNA) of the gene of interest was cloned downstream of these promoters in the same plasmid backbone. In the case of NPR-5, a 1.2-kb cDNA encoding sequence was custom synthesized (GeneArt, Thermo Fisher Scientific GENEART GmbH, Germany) and cloned in pPD95.75 utilizing KpnI and XhoI restriction sites. The vector backbones used were obtained from Addgene and the *Prig-3*::GCaMP5 plasmid was a gift from Cori Bargmann’s laboratory. The co-injection markers used were P*unc-122*::GFP and P*myo-2*::mCherry (pCFJ90) from Addgene and were injected at a concentration of 10–15 ng/μl. Transgenic strains were generated by previously described microinjection techniques using 25–30 ng/μl of the plasmid to generate stable transgenic array lines of *C. elegans* (Mello and Fire 1995).

### Reversal assays

Reversal assays were performed using well-fed young adult hermaphrodite animals. The animals were scored for off food reversal frequency conditions on NGM plates without food. *C. elegans* were transferred from seeded plates to unseeded ones using an eyelash pick and halocarbon oil. They were allowed to crawl on the unseeded plate for 30 sec and transferred to another unseeded plate that served as the final assay plate (90-mm NGM plate). Following a 1-min unscored acclimation period, animals were scored for the number of reversals over the next 5 min (Zhao *et al.* 2003). We defined a reversal by an animal as a backward motion greater than or equal to its pharynx length. At least 12 animals were assayed for each genotype. The number of body bends per reversal was calculated and plotted as previously described in Bhardwaj *et al.* (2018).

### Fluorescence imaging and image quantification

Imaging of neuron-specific GLR-1::GFP puncta was performed using the P*rig*-3::HA::GLR-1::GFP line (Hoerndli *et al.* 2013). The imaging was performed using the Leica (Leica Microsystems, Wetzlar, Germany) TCS SP8 confocal microscope and the Zeiss (Carl Zeiss AG, Oberkochen, Germany) fluorescence microscope Axio Imager Z2 with an Axiocam MRm camera. All the imaging experiments were performed on young adult animals. The animals were placed on 2% agarose pads and paralyzed using 2,3 butanedione monoxime (BDM) (30 mg/ml) as previously described (Sieburth *et al.* 2005). The AVA cell body was located and GLR-1::GFP was imaged in the region of the AVA cell body. Images of the *C. elegans* ventral nerve cord were captured just posterior to the nerve ring using a 63× objective. A fixed region of interest (ROI) was used for all the images and fluorescence intensities along the axons or the cell bodies were calculated using Image J Fiji [National Institutes of Health (NIH), Bethesda] (Schindelin *et al.* 2012). The threshold was specified such that the puncta of specific size were visible, and it was set using the control sample. The same threshold was applied for all the images. The mean fluorescence intensity was plotted for each sample.

### Calcium imaging

Calcium imaging was performed using GCaMP5 expressed in AVA command interneurons (P*rig-3*::GCaMP5). Calcium transients were recorded in freely navigating young adult animals on a slide with an agarose pad as previously described (Faumont *et al.* 2011). We used an Olympus (Olympus Corporation, Shinjuku, Tokyo, Japan) IX73 inverted microscope (40× objective with 0.6 NA) fitted with a worm tracker (Applied Scientific Instruments, Eugene, OR), and a Rolera Thunder EMCCD camera (QImaging, Surrey, Canada). Videos were acquired at 10 frames per second with 100-msec exposure using ImageJ software. The analysis of AVA activity was performed as described previously (Kerr 2006). The videos were analyzed using Fiji ImageJ software. The ROI was drawn as a 25 × 25 pixel circle over the AVA cell body that was expressing GCaMP5. The measured value of fluorescence from the ROI was taken as F_meas_, which included fluorescence from sample as well as background fluorescence (F_bkg_). The background fluorescence was estimated by repositioning the same ROI at a nonfluorescing region of the video. Then the fluorescence (*F*) from the given ROI was estimated by subtracting the background fluorescence from the measured fluorescence value (*i.e.*, *F*= *F*_meas_−*F*_bkg_). The fluorescence value was estimated for each frame after 100 msec by manual repositioning of the ROI. Calcium transients during reversals were plotted as Δ*F*/*F*_0_, where Δ*F* is the change in fluorescence (*F*) from the baseline value (*F*_0_). Baseline fluorescence (*F*_0_) was the fluorescence value calculated from the same ROI when C. elegans moved in the forward direction. Calcium transients represent a general trend of activity change during reversals for each genotype. Calcium levels were measured as Δ*F*/*F*_0_ max, which represents the maximum change in Δ*F*/*F*_0_ during each event.

### Statistical analyses

All statistical analyses were performed and *P* values determined using GraphPad Prism V6. Experimental data are shown as mean ± SEM. The data sets were analyzed using one-way ANOVA and the *post hoc* Bonferroni’s multiple comparison test in Graph Pad Prism V6. The level of significance was set at *P* < 0.05.

### Data availability

Strains and plasmids are available upon request. Supplemental Tables S1 and S3 contain the information for all strains and plasmids used in this study. All videos used in this work are also available upon request. Supplemental material available at figshare: https://doi.org/10.25386/genetics.10247969.

## Results

### Mutants in *rig-3* show increased reversals

**Figure 1:**
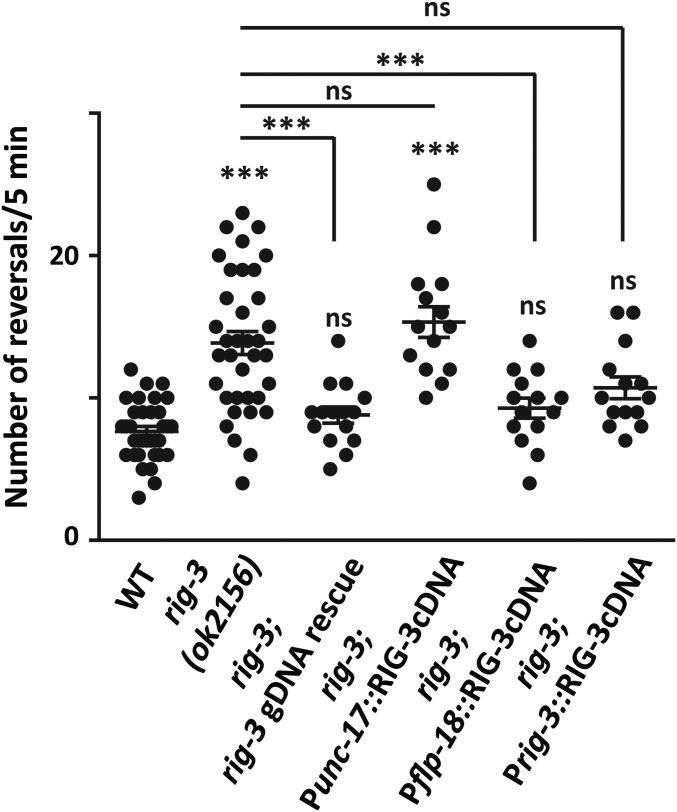
*rig-3* mutants show increased spontaneous reversal frequency. A dot plot indicating the results of reversal assays from wild-type (WT) control animals, *rig-3* mutants, and rescue lines expressing RIG-3 under its own promoter (genomic region of *rig-3*), in cholinergic neurons (P*unc-17* promoter), under the *flp-18* promoter expressed in multiple head neurons including the AVA interneuron, and under the *rig-3* promoter (P*rig-3* contains a 3-kb promoter region of *rig-3* and has been shown to largely express in the AVA interneuron and pharyngeal neurons). Reversals were counted manually from videos. In this dot plot, the number of dots represents the number of animals scored for the reversal assay. Each dot represents the number of spontaneous reversals per 5 min from one animal. The error bars represent ± SEM. Statistical significance was determined with one-way ANOVA using Bonferroni’s multiple comparison test. Significance is represented as *** *P* < 0.001 and “ns” for not significant. The statistics above each plot indicate significance with respect to the WT plot. The number of animals tested for each genotype is: WT (*n* = 34), *rig-3* (*n* = 36), *rig-3*; *rig-3* gDNA rescue (*n* = 15), *rig-3*; P*unc-17*::RIG-3 (*n* = 15), *rig-3*; P*flp-18*::RIG-3 (*n* = 15), and *rig-3*; P*rig-3*::RIG-3 (*n* = 14).

Studies have shown that RIG-3 plays an important role in maintaining acetylcholine receptor levels at the neuromuscular junction (Babu *et al.* 2011; Pandey *et al.* 2017). Apart from showing expression in cholinergic motor neurons, RIG-3 also shows expression in the anterior region that is thought to be restricted to the command interneuron AVA and pharyngeal neurons (Schwarz *et al.* 2009). Based on these previous reports, we hypothesized that RIG-3 could be affecting synaptic receptors in the AVA command interneuron to regulate signaling through the neural circuit that controls the reverse movement in *C. elegans*. We observed the reversal behavior of *rig-3** (**ok2156**)* mutant animals and found a significant increase in the reversal frequency of *rig-3* mutant animals when compared to WT control animals (Figure 1 and Videos 1 and 2). This observation indicated that RIG-3 could be modulating signaling through the reversal circuitry. We next went on to rescue this hyper-reversal phenotype using a construct containing the full-length *rig-3* genomic region, expressed in pharyngeal interneurons, AVA command interneuron, and cholinergic motor neurons (Babu *et al.* 2011). We found that this construct could rescue the increased reversal phenotype seen in the *rig-3* mutants (Figure 1). We further performed rescue experiments to pinpoint the site of action of RIG-3 function in the reversal circuitry. The neural circuit that controls the backward (reversal) movement in *C. elegans* has been well studied, and the role of three command interneurons, AVA, AVD, and AVE, has been established in this process (Chalfie *et al.* 1985; Gray *et al.* 2005; Piggott *et al.* 2011). RIG-3 expression has been shown in the cholinergic motor neurons along the body of the animals and in the AVA command interneurons in the head of the animal (Schwarz *et al.* 2009; Babu *et al.* 2011). We decided to attempt rescue of the *rig-3* hyper-reversal phenotype using *rig-3* cDNA expressed under the cholinergic promoter *unc-17* and the interneuron-specific promoter *flp-18* that is expressed in AVA, AIY, RIG, and RIM interneurons (Rogers *et al.* 2003; Kim and Li 2004; Williams *et al.* 2007). Previously, it has been shown that the expression of RIG-3 under the *unc-17* promoter was sufficient to rescue the increased acetylcholine receptor phenotype seen in the mutants (Babu *et al.* 2011). However, we found that RIG-3 expression using the *unc-17* promoter could not rescue the increased reversal phenotype seen in the mutants, while expressing RIG-3 under the *flp-18* promoter could largely rescue the hyper-reversal phenotype seen in the *rig-3* mutants (Figure 1). These results indicate that RIG-3 could be functioning at the level of interneurons to modulate reversal frequency in *C. elegans*. To further delineate the neurons through which RIG-3 could be functioning, we decided to rescue RIG-3 specifically using a 3-kb promoter upstream of RIG-3 that has previously been shown to express largely in AVA interneurons (Feinberg *et al.* 2008). Apart from the AVA interneuron, this promoter has been shown to have expression in the pharyngeal interneurons I1, I4, M4, and NSM (Schwarz *et al.* 2009; Bhardwaj *et al.* 2018). However, to our knowledge none of these pharyngeal interneurons has been shown to be involved in reversal behavior or have any direct synaptic connections with AVA, making rescue of reversal frequency using this promoter AVA dependent. Expressing RIG-3 using this promoter also appeared to bring reversal frequencies similar to WT levels in this line (Figure 1 and Video 3). It has been recently shown that AVA could be a cholinergic interneuron (Pereira *et al.* 2015). However, we found that expression of RIG-3 under the *unc-17* promoter could not rescue the increased reversal phenotype seen in the *rig-3* mutants giving rise to the possibility that the expression in AVA under the *unc-17* promoter could be lower or more variable in our array line and hence unable to rescue the reversal phenotype in the mutants. Together, these results suggest that RIG-3 functions in the AVA command interneuron to regulate reversal frequency in *C. elegans*.

### RIG-3 is required for maintaining GLR-1 receptor levels in the AVA command interneuron

**Figure 2:**
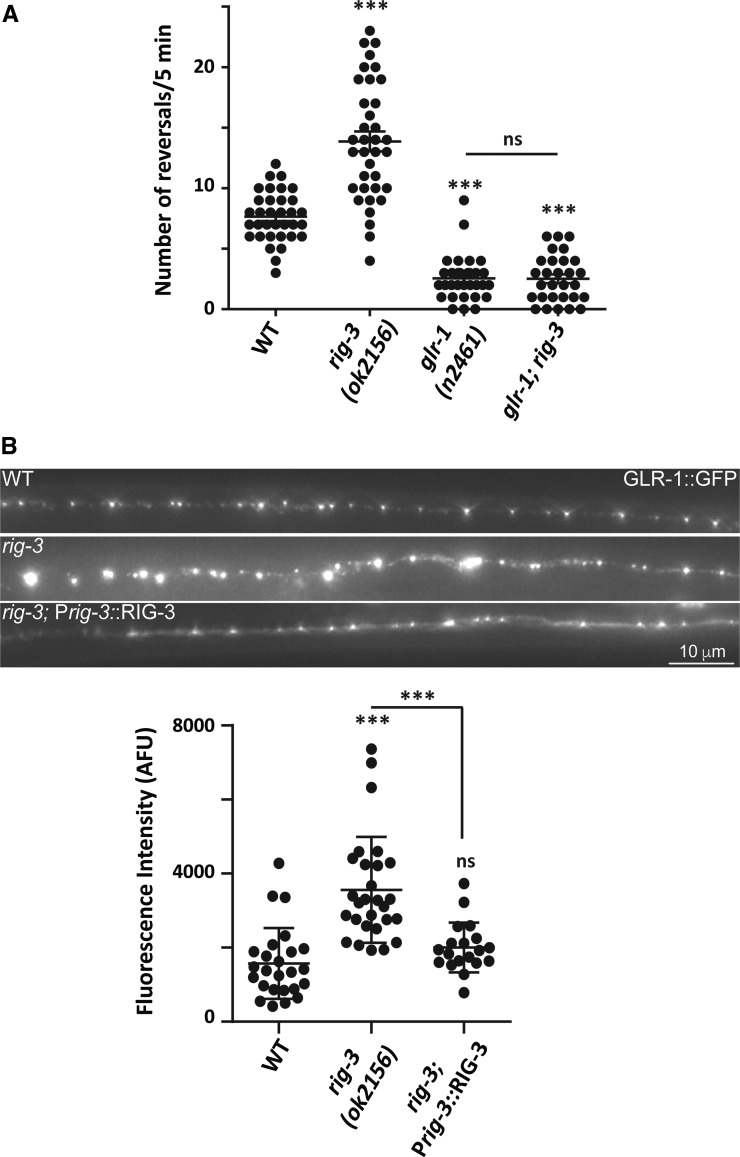
*rig-3* genetically functions through *glr-1*. (A) Reversal frequency represented as a dot plot from WT (*n* = 34), *rig-3* (*n* = 36), *glr-1* (*n* = 31), and *glr-1*; *rig-3* (*n* = 29) mutant animals. In this dot plot, the number of dots represents the number of animals scored for reversal assays. Each dot represents the number of spontaneous reversals per 5 min from one animal. The error bars represent ± SEM. Statistical significance was determined with one-way ANOVA with Bonferroni’s multiple comparison test. Significance is represented as *** *P* < 0.001 and “ns” for not significant. The statistics above each plot indicate significance with respect to the WT plot. (B) GLR-1::GFP puncta were imaged from the cord at the anterior region of AVA, just posterior to the nerve ring. The images on the top panel show GLR-1::GFP expression in WT (*n* = 25), *rig-3* mutants (*n* = 28), and *rig-3*; P*rig-3*::RIG-3 rescue line (*n* = 19). The dot plot in the bottom panel shows the quantitative measure of fluorescence intensity as an arbitrary fluorescence unit (AFU). Number of dots in the dot plot shows the number of animals imaged, where each dot represents the fluorescent intensity from a single animal. The error bars represent ± SEM. Statistical significance was determined with one-way ANOVA, Bonferroni’s multiple comparison test. Significance is represented as *** *P* < 0.001 and “ns” for not significant. The statistics above each plot indicate significance with respect to the WT plot.

Reversal behavior in *C. elegans* is dependent on the glutamate receptor GLR-1 (Hart *et al.* 1995; Zheng *et al.* 1999). Previous reports have suggested that reversal frequency is affected by changes in glutamatergic signaling. There are reports indicating that mutants showing decreased glutamatergic signaling, like *glr-1* and *eat-4*, show a significant reduction in reversal frequency (Zheng *et al.* 1999; Burbea *et al.* 2002). On the other hand, mutants with increased glutamatergic signaling show a significant increase in reversal frequency when compared with WT animals (Zheng *et al.* 1999; Burbea *et al.* 2002; Juo and Kaplan 2004; Schaefer and Rongo 2006; Juo *et al.* 2007). Based upon these studies, and the increased reversal frequency of *rig-3* mutants, we postulated that RIG-3 could be affecting glutamatergic signaling through GLR-1 receptors in the AVA command interneuron. If RIG-3 were to function through GLR-1, we hypothesized that the reversal frequency of *glr-1*; *rig-3* double mutants would be comparable to that seen in *glr-1* mutants. Upon testing the mutants, we found that those with mutations in *glr-1* (*glr-1* *(**n2461**)*) showed a significant decrease in reversal frequency when compared to WT animals as was previously reported (Juo *et al.* 2007; Figure 2A). Further, *glr-1*; *rig-3* double mutant animals showed similar reversal frequencies as were observed in *glr-1* mutants (Figure 2A). These data indicate that RIG-3-based regulation of reversal behavior could occur through GLR-1 receptors.

The possible requirement of RIG-3 in the AVA neuron to maintain reversal levels prompted us to look at the abundance of GLR-1 levels in this particular neuron. The AVA neuron is postsynaptic to 40 neurons, and it receives synaptic input from many of the sensory neurons, *i.e.*, ASH, AWC, ASE, AFD, ALM, and interneurons AIB and RIG, many of which are reported to be glutamatergic (White *et al.* 1986; Choi *et al.* 2015). Most of these postsynaptic connections are found near the nerve ring (White *et al.* 1986). The synaptic strength of these postsynaptic connections would depend upon the abundance of GLR-1 levels in the synapse near the nerve ring, which could further affect signaling through AVA. To determine whether RIG-3 regulates GLR-1 specifically in AVA, we used a previously described line where GLR-1 tagged with GFP (GLR-1::GFP) was expressed in the AVA interneuron using the *rig-3* promoter (Hoerndli *et al.* 2013). We analyzed the abundance of GLR-1::GFP puncta along the axon of the AVA neuron and at the cell body near the nerve ring in WT and *rig-3* mutants and found a significant increase in GLR-1::GFP levels along the axon and near the nerve ring in *rig-3* mutant animals when compared with WT controls (Figure 2B and Figure S1). We performed rescue for this increase in GLR-1::GFP of *rig-3* mutants by expressing RIG-3 under the AVA promoter *rig-3* and interneuron-specific promoter *flp-18* (Figure 2B and Figure S1).

Together these results suggest that RIG-3 functions through GLR-1 in the AVA command interneuron, where it could be regulating the abundance of GLR-1 receptors to maintain normal signaling across the reversal circuitry.

### Mutants in *rig-3* show increased AVA activity

**Figure 3:**
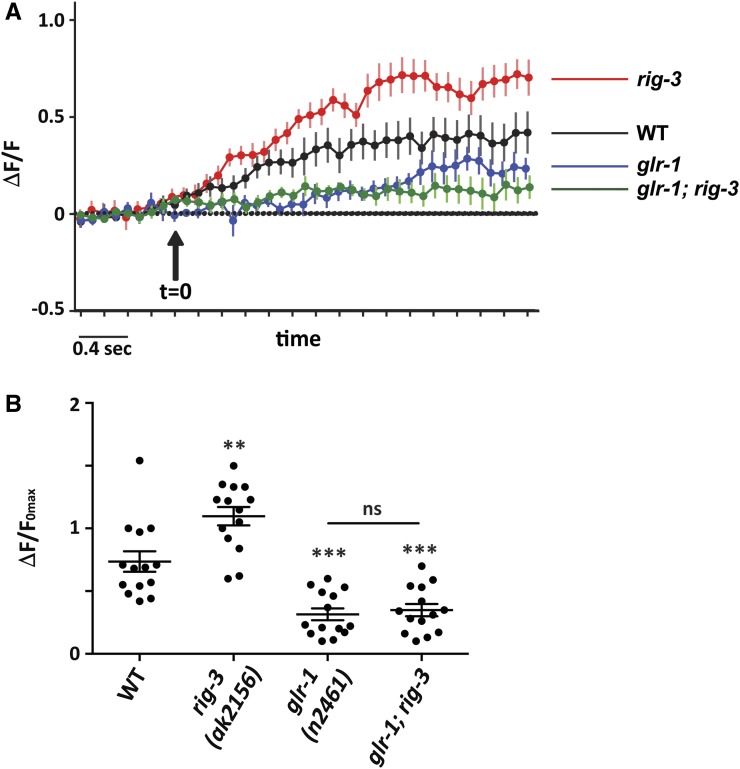
*rig-3* mutants show increased AVA activity during spontaneous reversals. (A) Average traces of calcium activity from freely reversing animals recorded using GCaMP5 expressed in the AVA command interneuron. The genotypes used include WT and mutant strains (*rig-3*, *glr-1*, and *glr-1*; *rig-3*). The arrow indicates the initiation of reversals at *t* = 0. Since reversals have variable durations, we have plotted here the shortest reversal for each genotype. The complete traces for all animals are shown in Figure S2A. (B) AVA activity represented as Δ*F*/*F*_0_ max that denotes the maximum transients in calcium levels during each reversal event. The number of dots in the dot plot represents the number of animals observed for calcium change, with each dot representing the maximum change in fluorescence from each *C. elegans*. Fourteen animals were imaged per genotype. The error bars represent ± SEM. Statistical significance was determined with one-way ANOVA with Bonferroni’s multiple comparison test. Significance is represented as ** *P* < 0.01, *** *P* < 0.001, and “ns” for not significant. The statistics above each plot indicate significance with respect to the WT plot.

The AVA command interneuron has been reported to be involved in reversal initiation, thus tracking the activity of the AVA neuron through *in vivo* calcium imaging during reversals could allow us to further understand changes in the phenotypes seen in *rig-3* mutants (Gray *et al.* 2005; Piggott *et al.* 2011). Previous studies suggest that there is an increase in the calcium levels in AVA during reversals (Piggott *et al.* 2011; Bhardwaj *et al.* 2018). This information along with our results showing increased abundance of GLR-1 levels in *rig-3* mutant animals suggest a possible increase in the synaptic strength of the AVA command interneurons in *rig-3* mutants (Figure 2B). These data prompted us to analyze the activity of AVA by tracking AVA activity in freely moving *rig-3* mutant animals. To determine the activity of AVA, we used a line expressing GCaMP5, a calcium sensor, under the 3-kb *rig-3* promoter (Larsch *et al.* 2013). Upon testing the activity of AVA, we found that *rig-3* mutants show a significant increase in AVA activity when compared with WT control animals (Figure 3, A and B, Figure S2A, and Videos 4 and 5). This result suggests that this increase in AVA activity could be the result of increased synaptic strength at AVA synaptic inputs, due to increased GLR-1 levels. A previous study suggested that glutamate-gated currents in AVA are largely dependent upon GLR-1 (Mellem *et al.* 2002). To further elaborate on the role of GLR-1 in this process, we decided to perform the calcium imaging experiments in *glr-1* mutants. We found significantly lower AVA activity in *glr-1* mutants when compared with WT animals (Figure 3, A and B, Figure S2A, and Video 6). This suggests that AVA activity is primarily determined by GLR-1 receptors. To further confirm that RIG-3 functions through GLR-1 to modulate the AVA activity, we did the calcium imaging of AVA in *glr-1*; *rig-3* double mutant animals. We found that the AVA activity in *glr-1*; *rig-3* mutants was comparable to that seen in *glr-1* mutants, and significantly less than WT control animals (Figure 3, A and B, Figure S2A, and Videos 6 and 7). These data support our previous results that RIG-3 functions through GLR-1 to modulate reversal behavior in *C. elegans*.

Our previous work has elaborated on the role of AVA activity and reversal length (Bhardwaj *et al.* 2018). Since *rig-3* mutants showed a significant increase in AVA activity in comparison with WT control animals, we went on to test for reversal length in these mutant animals. We have previously shown that mutants in *flp-18* show increased AVA activity and increased reversal length. We used *flp-18* animals as controls and found that unlike in *flp-18* mutants, *rig-3* mutant animals did not show increased reversal lengths (Bhardwaj *et al.* 2018; Figure S2B). This observation indicates that AVA activation may not be the only factor to control the length of the reverse movement. In our previous study, we reasoned that calcium-raising duration was also an essential factor to control the reversal length; in *flp-18* mutant *C. elegans*, we observed increased calcium-raising duration when compared to WT control animals (Bhardwaj *et al.* 2018). This calcium-raising duration was substantially lower in *rig-3* mutant animals when compared to *flp-18** C. elegans* (data not shown). This could be a possible reason for *rig-3* mutant animals showing early reversal termination that is comparable to that seen in WT *C. elegans*. We next wanted to understand the mechanism of increased reversals in AVA and tested the role of the FLP-18 neuropeptide that is released from AVA in this process.

### FLP-18 and NPR-5 signaling modulates reversal frequency

**Figure 4:**
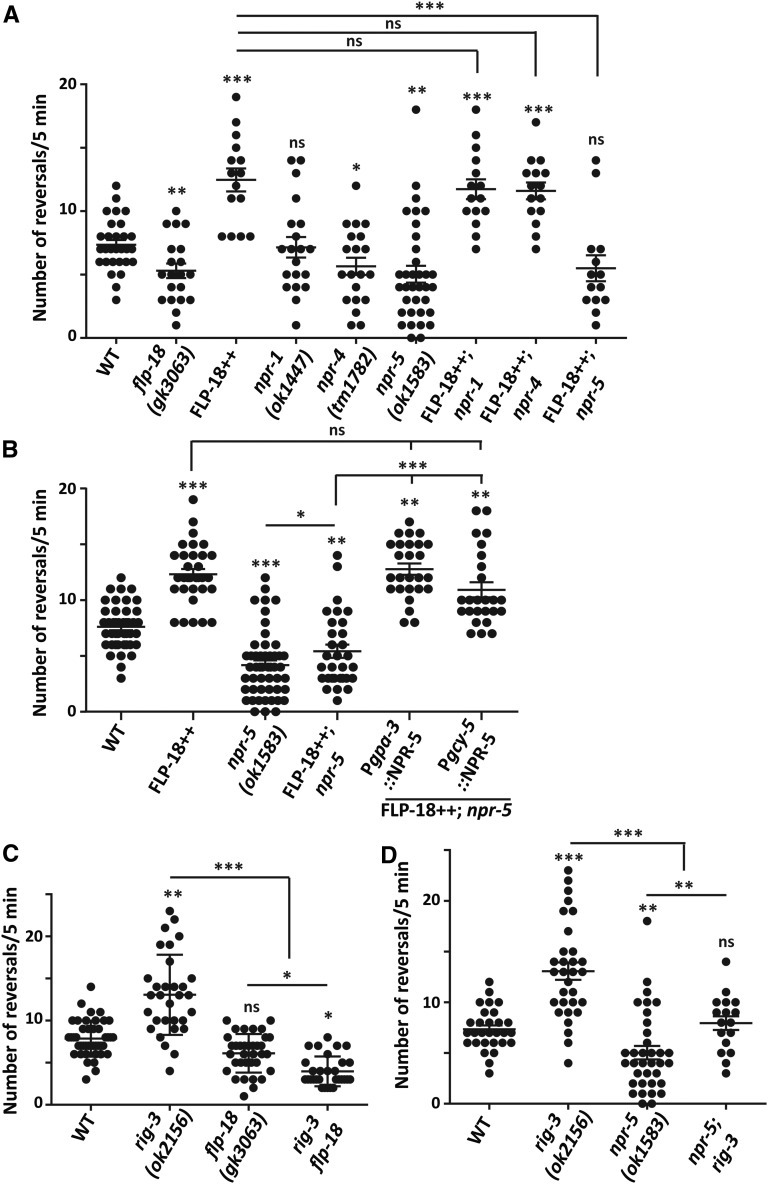
FLP-18 peptide regulates the spontaneous reversal initiation through the NPR-5 receptor. (A) Quantitation of reversal frequency from WT, *flp-18*, FLP-18++ (FLP-18 overexpression line), *npr-1*, *npr-4*, *npr-5*, and the three *npr* mutant lines with FLP-18++. The number of dots represents the number of animals scored, and each dot represents the number of reversals per 5 min in each animal of the same genotype in all graphs. The error bars represent ± SEM. Statistical significance was determined with one-way ANOVA with Bonferroni’s multiple comparison test. Significance is represented as * *P* < 0.05, ** *P* < 0.01, *** *P* < 0.001, and “ns” for not significant. The statistics above each plot indicate significance with respect to the WT plot. The number of animals tested for each genotype is: WT (*n* = 29), *flp-18* (*n* = 20), FLP-18++ (*n* = 15), *npr-1* (*n* = 20), *npr-4* (*n* = 20), *npr-5* (*n* = 35), FLP-18++; *npr-1* (*n* = 15), FLP-18++; *npr-4* (*n* = 15), and FLP-18++; *npr*-5 (*n* = 14). (B) Results of reversal assays depicted as a dot plot. The animals assayed were WT (*n* = 39), FLP-18++ (*n* = 25), *npr-5* (*n* = 45), FLP-18++; *npr-5* (*n* = 24), FLP-18++; *npr-5*; P*gpa-3*::NPR-5 (*n* = 25), and FLP-18++; *npr-5*; P*gcy-5*::NPR-5 (*n* = 24). The error bars represent ± SEM. Statistical significance was determined with one-way ANOVA with Bonferroni’s multiple comparison test. Significance is represented as * *P* < 0.05, ** *P* < 0.01, *** *P* < 0.001, and “ns” for not significant. The statistics above each plot indicate significance with respect to the WT plot. (C) Dot plot of reversals from WT (*n* = 43), *rig-3* (*n* = 31), *flp-18* (*n* = 35), and *rig-3* *flp-18* (*n* = 30) animals. The error bars represent ± SEM. Statistical significance was determined with one-way ANOVA with Bonferroni’s multiple comparison test. Significance is represented as * *P* < 0.05, ** *P* < 0.01, *** *P* < 0.001, and “ns” for not significant. The statistics above each plot indicate significance with respect to the WT plot. (D) A dot plot showing reversal frequency in WT (*n* = 29), *rig-3* (*n* = 31), *npr-5* (*n* = 35), and *npr-5*; *rig-3* (*n* = 17) mutants. The error bars represent ± SEM. Statistical significance was determined with one-way ANOVA with Bonferroni’s multiple comparison test. Significance is represented as ** *P* < 0.01, *** *P* < 0.001, and “ns” for not significant. The statistics above each plot indicate significance with respect to the WT plot.

Neuropeptides are small peptides that act as signaling molecules to allow communication between neurons; this communication can either be synaptic or extrasynaptic. Their extrasynaptic functioning allows them to modulate the activity of the entire neural circuit, which could ultimately modulate the behavioral output of the circuit (Li and Kim 2008). Thus, neuropeptides related to any circuit are of immense importance to study modulation of related behaviors. Our results so far suggest that the increased reversal frequency of *rig-3* mutants could be attributed to higher AVA signaling. These data led us to look at the signaling molecules released from AVA, which could be responsible for RIG-3 dependent modulation of reversal frequency. Neuropeptides FLP-1 and FLP-18 are two important signaling molecules released by the AVA command interneuron (Nelson *et al.* 1998; Rogers *et al.* 2003). These neuropeptides belong to the FMRFamide-like family that is reported to modulate several locomotion-dependent behaviors in *C. elegans* (Kim and Li 2004; Peymen *et al.* 2014; Chang *et al.* 2015). In previous reports, FLP-1 has been known to regulate coordinated sinusoidal movement of *C. elegans*, while FLP-18 has been shown to modulate reversal behavior in *C. elegans* (Nelson *et al.* 1998; Cohen *et al.* 2009; Bhardwaj *et al.* 2018). Based on previous work, we analyzed the reversal behavior in mutants of *flp-18* (*flp-18* (*gk3063*)) and found that they showed a small decrease in reversal frequency (Cohen *et al.* 2009; Bhardwaj *et al.* 2018; Figure 4A). This allowed us to postulate that FLP-18 could be an important molecule that could regulate reversal initiation based on the activity of AVA. To test this hypothesis, we tested the FLP-18 overexpression line, *i.e.*, P*flp-18*::FLP-18::SL2::GFP (Cohen *et al.* 2009), and found that these animals showed a significant increase in reversal frequency when compared to WT control animals (Figure 4A).

We next decided to identify the receptor through which FLP-18 could be functioning to modulate the reversal frequency. Previous work has shown that NPR-1, NPR-4, NPR-5, NPR-10, and NPR-11 could act as receptors for FLP-18 (Li and Kim 2014). The expression patterns for these receptors except that of NPR-10 have been well documented. NPR-1 is expressed in sensory and motor neurons, NPR-4 is largely expressed in the AVA interneuron, and NPR-5 and NPR-11 expression is reported to be mainly in sensory neurons (de Bono and Bargmann 1998; Wang and Wadsworth 2002; Cohen *et al.* 2009; Chalasani *et al.* 2010). To delineate the receptor through which FLP-18 modulates the reversal frequency, we started observing the reversal frequencies in mutants of the FLP-18 receptor genes: *npr-1* (*ok1447**)*, *npr-4* (*tm1782*), and *npr-5* (*ok1583*). We found that the reversal frequency in *npr-1* mutants was comparable to WT control animals, whereas *npr-4* and *npr-5* mutant animals showed a decrease in reversal frequency in comparison to WT animals (Figure 4A). These results suggested that NPR-4 and/or NPR-5 could be receptors for FLP-18 that allow for regulation of reversal frequency in *C. elegans*. To further elucidate the candidate receptor for FLP-18, we generated double mutants of the FLP-18 overexpression (FLP-18++) line with each receptor mutant, *i.e.*, *npr-1*, *npr-4*, and *npr-5*. We found that the FLP-18++ line with *npr-5* mutants showed a significant decrease in reversal frequency when compared with the FLP-18++ line alone (Figure 4A). Moreover, the FLP-18++ line with *npr-1* and *npr-4* mutants showed a phenotype similar to what was observed in the FLP-18 overexpression line by itself (Figure 4A). These results suggest that FLP-18/NPR-5 signaling could be important in the modulation of reversal initiation behavior.

We then tried to identify the neurons in which NPR-5 could be functioning by performing rescue experiments. We initially rescued the FLP-18++; *npr-5* phenotype by expressed NPR-5 largely in sensory neurons and in the AIZ and PVT interneurons using the *gpa-3* promoter (Zwaal *et al.* 1997 and wormbase.org). This experiment indicated that expression of NPR-5 largely in sensory neurons completely rescued the suppression of reversals seen in the FLP-18++; *npr-5* mutant animals (Figure 4B). We next expressed NPR-5 in the ASER sensory neurons using the *gcy-5* promoter (Yu *et al.* 1997; Chang *et al.* 2003). We again found that expression of NPR-5 in the ASER sensory neuron could also rescue the suppressed reversals seen in the FLP-18++; *npr-5* strain of *C. elegans* (Figure 4B). To rule out the possibility that overexpression of NPR-5 in the rescue lines could cause changes in basal reversal frequency, we went on to use these rescue lines to rescue the reversal frequency defects seen in *npr-5* mutants and found that both lines could rescue the decreased reversals seen in the *npr-5* mutants, and neither rescue lines showed the increase in reversal frequency that was comparable to the FLP-18++ strain (Figure S3, A and B). These data indicate that NPR-5 could be functioning in ASER to maintain reversal frequency in *C. elegans*. However, these data do not preclude the functioning of NPR-5 in other neurons to maintain reversal initiation.

We next went on to test if RIG-3 could be functioning through the FLP-18/NPR-5 signaling pathway to modulate reversal frequency. To perform this experiment, we decided to test the reversal frequency *rig-3* *flp-18* and *npr-5*; *rig-3* double mutant animals. We found a significant decrease in reversal frequency in both the *rig-3* *flp-18* and the *npr-5*; *rig-3* double mutant animals when compared with *rig-3* mutant animals (Figure 4, C and D). Finally, to test if the increased reversals in the FLP-18 overexpression line is dependent on GLR-1 levels we tested the reversals in the FLP-18++; *glr-1* line and found a significant reduction in reversal frequency in this line in comparison with the FLP-18++ line (Figure S3C). Together, our results suggest that RIG-3/FLP-18/NPR-5 could play a role in maintaining reversal initiations in *C. elegans*.

## Discussion

*C. elegans* initially search for food locally in a process that is dependent upon local search/exploratory behaviors. The extent of this local search is largely indicated by the frequency of reverse movements (Zhao *et al.* 2003; Gray *et al.* 2005). Multiple genetic screens have found mutants that alter the reversal frequency in *C. elegans* (Rongo *et al.* 1998; Rongo and Kaplan 1999; Zheng *et al.* 2004, 2006). In this study we have identified RIG-3, an IgSF protein, as an important regulator of reversal behavior in *C. elegans*.

RIG-3 is expressed in AVA command interneurons and a small number of pharyngeal interneurons as well as in cholinergic motor neurons (Schwarz *et al.* 2009; Babu *et al.* 2011). Previous studies have also shown that RIG-3 functions at the neuromuscular junction, where it has been reported to regulate the acetylcholine receptor delivery (Babu *et al.* 2011; Pandey *et al.* 2017). Our results suggest that animals lacking *rig-3* show more frequent spontaneous reversals in comparison to WT control animals (Figure 1). Previous work has shown that the AVA command interneuron acts as a master controller of reversal movement (Piggott *et al.* 2011). Any signaling defect through AVA could result in defects in reversal response. Our results further elaborate the fact that RIG-3 functions in the reversal circuitry to allow for normal reversal initiation.

In *C. elegans*, increased reversal frequency can be directly related to the extent of glutamatergic signaling through the reversal circuitry. Increased GLR-1 receptors allow the animals to reverse more while mutants in *glr-1* showed a significant decrease in reversal frequency (Zheng *et al.* 1999; Burbea *et al.* 2002; Juo and Kaplan 2004; Schaefer and Rongo 2006; Juo *et al.* 2007). Increased reversal frequency in *rig-3* mutant animals allowed us to hypothesize that RIG-3 could be functioning through GLR-1 receptors to modulate reversal behavior. We found that *rig-3* mutants showed increased GLR-1 levels in the AVA command interneuron (Figure 2). In the AVA command interneuron, most of the synaptic inputs are near the nerve ring (White *et al.* 1986). Hence, increased GLR-1::GFP levels in this region suggest increased strength of AVA synaptic inputs, which could be responsible for increased signaling through AVA.

To confirm the increased signaling through AVA, we carried out calcium imaging in the AVA command interneuron in freely moving animals and found a significant increase in AVA calcium transients in *rig-3* mutants as compared to WT control animals (Figure 3). These results suggest increased AVA activity in *rig-3* mutants, which further supports our hypothesis that there could be increased synaptic strength of inputs to AVA in *rig-3* mutants. In a previous study by us we have characterized the calcium transients of AVA with respect to reversal length, where we showed changes in calcium levels with reversal length and duration of AVA activation. That study suggested that prolonged activation of AVA interneurons could be allowing the animal to execute longer reversals by minimizing the chance of execution of a second reversal event (Bhardwaj *et al.* 2018). In this study the change in AVA calcium levels is significantly more than that in WT for a very short duration at the start of reversals. However, this change in calcium levels in *rig-3* mutants is considerably less than that seen in *flp-18* mutants. Our data suggest that *rig-3* affects signaling of AVA in a short duration, which could be sufficient to initiate its motor output (reversal), but there is no prolonged activation of AVA in *rig-3* mutants as seen in *flp-18* mutants. The other important outcome of the calcium imaging experiments was noting a significant decrease in calcium transients of AVA in *glr-1* mutants (Figure 3). This further supports previous work that has reported decreased GLR-1-dependent AVA currents in *glr-1* mutants (Mellem *et al.* 2002). Further, double mutants of *glr-1*; *rig-3* also showed similar calcium transients as was observed in *glr-1* mutants validating our finding that RIG-3 likely functions through GLR-1 to modulate AVA activity, which could in turn result in altered reversal behavior in *C. elegans*.

**Figure 5:**
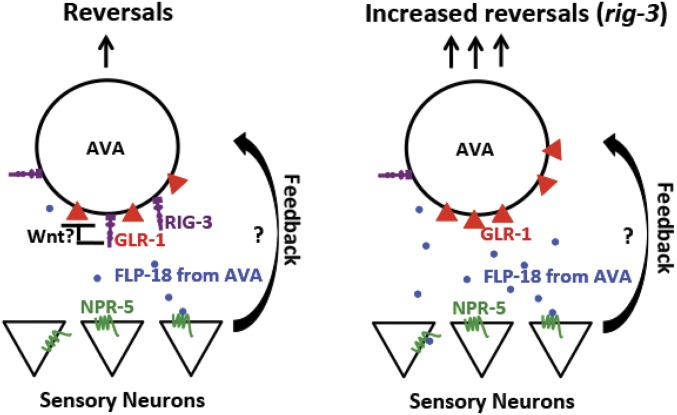
Possible model of RIG-3 modulating AVA function. The model indicates the function of RIG-3 in the AVA interneuron. Loss of RIG-3 shows increased GLR-1 levels; however, the mechanism of how RIG-3 affects GLR-1 is still unknown. Upon activation of AVA, FLP-18 is released and functions through NPR-5 in sensory neurons, which in turn affect reversals. How NPR-5 affects reversal frequency is another unknown in the model.

Taken together our data suggest a model wherein RIG-3 appears to function in AVA to regulate both glutamate receptor levels in AVA and as a consequence the activity of AVA (illustrated in Figure 5). This work has not uncovered the mechanism of how RIG-3 might affect GLR-1 levels in AVA. A possible mechanism could involve maintaining receptor levels by affecting the delivery or anchoring of receptors, as RIG-3 is a cell surface IgSF molecule. This is in contrast to previous work on RIG-3 that has shown that RIG-3 in cholinergic neurons affects receptor levels in the body-wall muscle in a non-cell-autonomous manner through the WNT signaling pathway (Babu *et al.* 2011; Pandey *et al.* 2017). It will be interesting to test if RIG-3 functions through the WNT signaling pathway to maintain GLR-1 levels in AVA.

These results prompted us to delve further into the molecular basis of RIG-3-dependent modulation of reversal frequency. In previous studies, neuropeptides have been reported to be involved in modulation of locomotory circuits in *C. elegans* (Li and Kim 2014). FLP-1 and FLP-18 are two main neuropeptides that are released from AVA and are known to affect the locomotory circuit in *C. elegans*. FLP-1 has been reported to affect locomotory circuits to modulate normal sinusoidal movement in *C. elegans* and mutants of *flp-1* show movement defects, while FLP-18 has been shown to affect the reversal behavior of *C. elegans* (Nelson *et al.* 1998; Cohen *et al.* 2009; Bhardwaj *et al.* 2018). Mutations in *flp-18* show decreased reversal frequency when compared with WT control animals (Cohen *et al.* 2009; Bhardwaj *et al.* 2018). We also observed a significant increase in reversal frequency in a FLP-18 overexpression line, and this phenotype was suppressed by the *npr-5* mutation (Figure 4, A and B). Together, these data provide strong evidence that FLP-18 acts through NPR-5 to regulate reversal frequency. The data also suggest that NPR-5 could be functioning in sensory neurons to mediate the role of FLP-18 from interneurons. How NPR-5 could be working to modulate reversals from sensory neurons would be an interesting question. Finally, to test if there is a connection between RIG-3/GLR-1 and the FLP-18/NPR-5 signaling pathways we studied the suppression of the *rig-3* mutant phenotype with the *flp-18* and *npr-5* mutations as well as the suppression of the FLP-18 overexpression phenotype with the *glr-1* mutation. In all three cases we found suppression of the increased reversals phenotype (Figure 4, C and D). These data allow us to speculate that RIG-3 could be acting through FLP-18/NPR-5 (illustrated in Figure 5). However, these data do not preclude the possibility that RIG-3 and NPR-5 act in separate parallel pathways or that RIG-3 acts through NPR-5 as well as through other molecules to maintain normal reversal frequency during local search behavior in *C. elegans*.
